# A General Model of Ion Passive Transmembrane Transport Based on Ionic Concentration

**DOI:** 10.3389/fncom.2018.00110

**Published:** 2019-01-22

**Authors:** Vincent Qiqian Wang, Shenquan Liu

**Affiliations:** School of Mathematics, South China University of Technology, Guangzhou, China

**Keywords:** membrane, ion channel, gate, filter, ionic concentration and flux, electrophysiology, neurosciences

## Abstract

Current mainstream neural computing is based on the electricity model proposed by Hodgkin and Huxley in 1952, the core of which is ion passive transmembrane transport controlled by ion channels. However, studies on the evolutionary history of ion channels have shown that some neuronal ion channels predate the neurons. Thus, to deepen our understanding of neuronal activities, ion channel models should be applied to other cells. Expanding the scope of electrophysiological experiments from nerve to muscle, animal to plant, and metazoa to protozoa, has lead the discovery of a number of ion channels. Moreover, the properties of these newly discovered ion channels are too complex to be described by current common models. Hence this paper has presented a convenient method for estimating the distribution of ions under an electric field and established a general ionic concentration-based model of ion passive transmembrane transport that is simple but capable of explaining and simulating the complex phenomena of patch clamp experiments, is applicable to different ion channels in different cells of different species, and conforms to the current general understanding of ion channels. Finally, we designed a series of mathematical experiments, which we have compared with the results of typical electrophysiological experiments conducted on plant cells, oocytes, myocytes, cardiomyocytes, and neurocytes, to verify the model.

## 1. Introduction

It is well-established that ion channels and pumps generate ion transmembrane transport. The classic models of passive transport mechanisms were derived from the study of neurons, which were considered to be an electric capacitor. The most famous and earliest model, which depended on this idea, was designed by Hodgkin and Huxley (1952). However, in the biological sense, neurons are far from being an electric capacitor. In fact, electrophysiological phenomena are common in all types of cells; currents and potentials have been recorded in myocytes [e.g., denervated frog muscle fibers (Neher and Sakmann, 1976)] and plant cells [e.g., xylem parenchyma of barley roots (Wegner and Raschke, 1994)]. It is inappropriate to think of these cells as capacitors. The so-called process of cell charging, in fact, is the cationic entry into the cell, or the anion leaving; the discharge process is opposite. Thus, a cell is more like an ion container than a capacitor.

If cells are not thought of as capacitors, how should membrane potential and membrane current be read? In fact, membrane potential is a measure of electric charge density, generated by intracellular and extracellular ion distribution. When the internal positive and negative charge are not equal, owing to the electric field force generated by the internal ions which is perpendicular to the membrane, the charged ions inside and outside the cell will approximate to or deviate from the membrane surface, resulting in the non-uniformed distribution of ions.

In addition to the electric fields generated by the internal ions, people can stimulate neurons using an outer artificial electric field. Magnetic stimulation can also stimulate neurons (Walsh and Rushworth, 1999). For example, placing a transcranial magnetic stimulation coil above the skull over a region of interest, when a changing electric current flows through the coil, an electromagnetic field is created (Polson et al., 1982). According to Faraday's law, this induces an electric field that can stimulate cortical neurons (Pashut et al., 2011). However, experimental studies have shown that neurons are insensitive to transverse outer electric field stimulation (Reilly, 1989). Therefore, when we focus on transmembrane ion transport which is perpendicular to the membrane, we can ignore the outer electromagnetic field and consider only the electric field generated by the ions.

For these reasons, although we cannot use the Nernst equation to calculate the membrane potential directly using the ionic concentration before estimating the ion density on the membrane surface, we may still borrow some ideas from the study of the electrical double layer. The electrical double layer is an array of charged particles and oriented dipoles that is thought to exist at every interface (Grahame, 1947). Extending this idea to both sides of the membrane, we can estimate the ion density on the membrane surface. Then the membrane potential measured experimentally, can be calculated numerically as a function of the ion density on the membrane surface. Membrane current is the sum of the charges carried by the transmembrane ion flux. The stimulation current, in fact, generates a redox reaction, and creates an ionic flow to stimulate the membrane.

The classic ion channel model (Hodgkin and Huxley, 1952) was constructed based on the measurement of current-voltage relationships in the membrane of the giant axons of the Loligo (a genus of squid) (Hodgkin et al., 1952) using a voltage clamp for voltage-gated ion channels. Voltage-gated ion channels have been studied thoroughly because it is convenient to measure the electric current while controlling the voltage with a patch clamp. It is generally acknowledged that voltage-gated ion channels are responsible for the electrical activity in a variety of animal cell types; however, voltage-gated ion channels are also found in prokaryotes and diverse range of eukaryotes, and probably exist in all life forms. Furthermore, multicellular animals evolved more than 650 million years ago, whereas nervous systems and muscles evolved much later. For instance, it has been shown that voltage-gated sodium channels predate neurons (Zakon, 2012), like many other neuronal channels and receptors. Therefore, although the voltage-gated ion channels play a major role in the electrical excitability of nerve cells, they have been in existence before nerve system appeared. It is generally believed that voltage-gated ion channels evolved from a prokaryote 2-TM channel. Following the addition of four more domains, an early, ligand-gated, 6-TM protein gave rise to the voltage-gated potassium family and extant ligand-gated potassium channels. Then, following two rounds of gene duplication, this ligand-gated potassium channel formed a four-domain 6-TM channel, such as calcium and sodium channels (Anderson and Greenberg, 2001).

Thus, it is likely that ion channels were created for other purposes. For example, voltage-gated potassium channels often play a role in the depolarization of nerve cells; yet this channel appeared three billion years ago in bacteria and occurs in all organisms (Zakon, 2012). In plants, potassium is mainly absorbed by the roots, which serves an essential role as an osmoticum. Moreover, the potassium channels of root cells, which mediate potassium uptake, play an important role in regulating ion concentration (Hirsch et al., 1998). Some scholars have tried to model potassium uptake by cells via potassium channels (Dreyer, 2017), which is valuable. Therefore, it is not possible to grasp the nature of voltage-gated ion channels by means of electrical signal generation and conduction alone.

Hence, we should realize that the model of ion passive transmembrane transport, which is not universally applicable to different ion channels in the different cells of different species, is insufficient to describe the nature of neural activities. In this paper, we propose a general model.

## 2. Foundation

### 2.1. Operators

In this paper, we use some operation symbols for sequences to simplify the expressions: (*a*_*i, j*_)·(*b*_*i, j*_) = (*a*_*i, j*_·*b*_*i, j*_), (*a*_*i*_)·(*b*_*i, j*_) = (*b*_*i, j*_)·(*a*_*i*_) = (*a*_*i*_·*b*_*i, j*_), (*a*_*i*_)·(*b*_*i*_) = (*a*_*i*_·*b*_*i*_); ${\left(a_{i}\right)}^{\cdot \left(b_{i}\right)}=\left(a_{i}^{b_{i}}\right)$, ${\left(a_{i}\right)}^{\cdot b}=\left(a_{i}^{b}\right)$; $\left(a_{i,j}\right)⊘\left(b_{i,j}\right)=\left(\frac{a_{i,j}}{b_{i,j}}\right)$, $\left(a_{i}\right)⊘\left(b_{i}\right)=\left(\frac{a_{i}}{b_{i}}\right)$; $\sum \left(a_{i}\right)=\underset{i}{\sum }a_{i}$, $\sum \left(a_{i,j}\right)=\left(\underset{i}{\sum }a_{i,j}\right)$.

### 2.2. Ionic Flux

Electrical signals of nerve are mainly generated by the flow of charged ions. This paper mainly discusses transmembrane ion flow. Thus, spatially, we only consider the direction perpendicular to the membrane surface. The ion flow considered in this paper is mainly caused by diffusion and electromigration,

(1) $$\text{J}={\text{J}}_{\text{d}}+{\text{J}}_{\text{e}},$$

where ***ȷ*** = (ȷ_*i*_), ȷ_*i*_ is the flux density of ion *i*, abbreviated as *ionic flux* in this paper. Ions diffuse from high concentration to low, and it is well-known that

(2) $${\text{J}}_{\text{d}}=-D\circ \frac{\partial c}{\partial x},$$

where **D** = (*D*_*i*_), *D*_*i*_ is the diffusion coefficient of ion *i*, and **c** = (*c*_*i*_), *c*_*i*_ is the concentration of ion *i*. The uncommon operational symbols in this paper are explained above. In solution, the velocity of ionic electromigration is generally considered to be proportional to the electric field intensity ϵ, so the ionic flux of electromigration

(3) $${\text{j}}_{\text{e}}=\epsilon \;\cdot \;c\circ z\circ \mu ,$$

where ***μ*** = μ_*i*_, $\mu_{i}=\frac{D_{i}F}{RT}$ is experiential, *R* is the molar gas constant, *F* = *eN*_*A*_ is the Faraday constant, *T* is the temperature (with a default of 290 K in this paper), and **z** = (*z*_*i*_), *z*_*i*_ is the valence of the ion *i*. It is difficult to measure ionic flux directly, but easy to measure the current generated by the ions flowing and the redox reaction. The current density (hereafter referred to as *current* for short) generated by ionic flux is

(4) ı=F·z·j.

Therefore, it is easy to calculate the current from ionic flux, but difficult to calculate ionic flux from the current. When the cell membrane is referred to as being given a stimulation current *ı*_s_, the current is not directly stimulating the membrane, but is involved in the redox reaction on the surface of the electrode, which generates an ionic flux that we refer to as *stimulation ionic flux* in this paper. There are several common electrodes, for the sake of convenience, here we choose silver electrodes (Ag/AgCl), on whose surface, the redox reaction is

(5) $$\text{AgCl}+\text{e}↔\text{Ag}+{\text{Cl}}^{-}.$$

The model would be too complicated if we considered the pipette solution. So, ignoring the interspace of the pipette, from Equations 4 and 5, the stimulation ionic flux can be estimated by

(6) $${\text{j}}_{s}{}_{i}=\{\begin{array}{ll} -\frac{1}{F}\cdot l\text{s} & \left(i={\text{Cl}}^{-}\right)\\ 0 & \left(i\neq {\text{Cl}}^{-}\right) \end{array}$$

where *ı*_s_ is the intensity of stimulus current.

### 2.3. Ionic Concentration

How ionic flux is estimated by ionic concentration and electric field has been given above. Since, in this case, the electric field is generated by the ions, the ion flux can be estimated by the ionic concentration distribution. A membrane, in any shape, separates a solution into two regions: internal and external. Thus, the morphology of the membrane affects the distribution of ions intracellular and extracellular. There are various shapes of cells, such as sphere, polyhedra, spindle and cylinder. For convenience, the shapes are generally simplified, usually to cylindrical and spherical shapes.

#### 2.3.1. Cylinder

It is tractable for mathematical analysis to reduce the complex branching structure of a dendritic tree to a simple cylinder (Holmes, 2015). In 1962, Rall showed that dendritic morphology could be reduced to a simple cylinder (Rall, 1962). This method was then applied to the study of axons. Roll the membrane into a cylinder, and let the inner surface of the membrane face inward and the outer surface face outward. Assume that the cylinder is standard, of which the length is long enough, and the ion distribution depends only on the distance to the axis of the cylinder. Then the rate of change of ionic concentration with time depends on ion flux,

(7) $$-\frac{\partial }{\partial t}c=(\frac{1}{x}+\frac{\partial }{\partial x})\text{J},$$

where *x* is the distance to the axis of the cylinder, at *x* = *r* is the membrane, if for any *x* the ionic distribution is uniform, ignoring the thickness of the membrane. If ignore the outer electromagnetic field, then the electric field is generated by the ions and is perpendicular to the membrane surface,

(8) $$\epsilon (y)=\frac{z}{y\varepsilon (y)}\cdot F\text{​}\int_{0}^{\;y}\text{​}\text{​}c\cdot x\text{ d}x,$$

where ε is the permittivity.

#### 2.3.2. Sphere

Since isolated cells in cultures or suspensions often have a spherical shape (Drasdo and Hohme, 2005), modeling a single cell as a sphere is also common, especially for electrophysiological experiments (Debruin and Krassowska, 1999). Packaged the membrane into a sphere, and also let the inner surface of the membrane face inward and the outer surface face outward. Assume that the sphere is standard, and the ion distribution depends only on the distance to the center of the sphere. Then the rate of change of ion concentration with time depends on ion flux,

(9) $$-\frac{\partial }{\partial t}c=(\frac{2}{x}+\frac{\partial }{\partial x})\text{J},$$

where *x* is the distance to the center of the sphere, at *x* = *r* is the membrane, if for any *x* the distribution of ions is uniform, ignoring the thickness of the membrane. If we ignore the outer electromagnetic field, then the electric field is generated by the ions and is perpendicular to the membrane surface,

(10) $$\epsilon (y)=\frac{z}{y^{2}\varepsilon (y)}\cdot F\text{​}\int_{0}^{\;y}\text{​}\text{​}c\cdot x^{2}\text{ d}x.$$

### 2.4. Ionic Density on Surface

To simulate whole cell patch clamp method, focusing on transmembrane transport, the sphere is more appropriate.

#### 2.4.1. Internal

The concentration of the solution in the experiment is, in general, the average concentration. The average internal ion concentration can be written as

(11) $$A_{\text{in}}c=3r^{-3}\text{​}\int_{0}^{\;r}\text{​}\text{​}c\cdot x^{2}\text{ d}x,$$

and A_in_*c*_*i*_ is traditionally written as [*i*]_in_. Transmembrane ion flow makes a small but significant change in the average internal ionic concentration that

(12) $$\frac{\text{d}}{\text{d}t}A_{\text{in}}c=3r^{-1}\cdot \text{u},$$

where **u** = −**ȷ**_*x* = *r*_ is the *transmembrane ion influx* (then *u* < 0 means outflow, as a salute to the classical model). In practical application, the change of average internal ion concentration can be directly calculated by transmembrane ion flux, instead of integrating. Set

(13) $$Qc=r^{-2}\text{​}\int_{0}^{r}\text{​}c\cdot x^{2}\text{ d}x$$

to describe the total amount of intracellular ions. Then the internal charge (per unit area) *Q* can be calculated by internal ionic concentration,

(14) Q=F·zQc,

of which the electric field derived from Equation 10 is

(15) $$\epsilon (r)=F\varepsilon^{-1}\cdot zQc.$$

Additionally, in practical application, the change in internal charge *Q* can be directly calculated by transmembrane ion flux ***u***,

(16) $$\frac{\text{d}}{\text{d}t}Q=F\cdot uz,$$

instead of integrating. Thus, the ion density on the inner surface of the membrane, referred to as *internal ionic density* for short, can be calculated by

(17) $$A_{\text{in}}c=r^{-2}\int_{r-\lambda }^{\;r}\text{​}\text{​​​​}c\circ x^{\circ 2}\circ \text{d}x,$$

where **λ** = (λ_*i*_), λ_*i*_ is the diameter of the ion *i* (see Appendix).

#### 2.4.2. External

For [0, *r*) is the internal space, (*r*, ∞) is external. The *average external ion concentration* is

(18) $$A_{\text{ex}}c=\underset{y\to \infty }{\lim}\frac{3}{y^{3}-r^{3}}\int_{r}^{\;y}\text{​}\text{​}c\cdot x^{2}\text{ d}x,$$

and A_ex_*c*_*i*_ is traditionally written as [*i*]_ex_, which is constant when the external space is large enough. At the same time, the ion density on the outer surface of the membrane, named *external ionic density* for short, should be

(19) $$T_{\text{ex}}c=\frac{1}{r^{2}}\int_{r}^{\;r+\lambda }c\circ x^{\circ 2}\circ \text{d}x.$$

#### 2.4.3. Estimating

Borrowing from the theory of *electrical double layer* (Grahame, 1947), the internal (external) solution could be thought to consist of two layers of ions: one of adsorbed ions and the other consisting of an ionic atmosphere. Thus, the membrane in contact with both the internal and external solution actually forms four layers. If the net charge is tiny, |***z*** Q***c***|≪ΣQ***c***, it can be derived from the equations in *Ionic Concentration* so that the net charge is very close to the membrane and is regarded as being adsorbed on the membrane surface, while the diffuse layer is relatively stable. Therefore, $\frac{{ȷ}_{|x-r|>\lambda_{i}}}{{ȷ}_{x=r}}\approx 0$ or ȷ≡0, as

(20) $$\frac{\partial c_{i}}{c_{i}\partial x}\approx \frac{\epsilon Fz_{i}}{RT},$$

for |*x*−*r*| < λ_*i*_. Thus, in the actual operation, a simple and convenient arithmetic can be used to estimate the membrane ionic density:

(21) $$T_{\text{in}}c\approx \lambda \circ A_{\text{in}}c\circ \left(1-\chi \;z\;Q\;c\;\cdot \lambda \circ z\right)⊘\left(1+\chi z\;Q\;c\;\cdot \;\lambda \circ z\right),$$

(22) $$T_{\text{ex}}c\approx \lambda \circ A_{\text{ex}}c\circ \left(1-\chi \;z\;Q\;c\;\cdot \lambda \circ z\right)⊘\left(1+\chi z\;Q\;c\;\cdot \;\lambda \circ z\right),$$

where

(23) $$\chi =\frac{F^{2}}{2RT\varepsilon_{\text{m}}}.$$

After measuring the membrane permittivity ε_m_ = ε(*r*), the membrane ionic density can be estimated.

### 2.5. Membrane Potential

In contact with the internal and external solution, the redox reaction on the electrode produces a potential difference, that is the membrane potential which is measured. The Nernst Equation is usually used to estimate the voltage, when the electrostatic force is absent, as ϵ = 0. However, it is also common that the internal ions almost inevitably generate an electric field force, which causes the ions to attach or leave the membrane surface. Actually, the membrane potential depends on the membrane ionic density,

(24) $$V_{\text{m}}=V\left(\begin{array}{c} T_{\text{in}}\\ T_{\text{ex}} \end{array}\right)c.$$

where

(25) $$V\left(\begin{array}{c} a\\ b \end{array}\right)\;=\frac{RT}{F}⊘z\cdot \text{ ln }(a⊘b).$$

Therefore, after estimating the ionic density on the membrane, we could calculate the membrane potential difference under the electric field.

It can be seen that dependence on membrane potential is essentially a special case of dependence on membrane ion density.

In the whole cell patch clamp experiment, the pipette is connected to the intracellular fluid. Then, the difference between the stimulus voltage and the membrane potential produces a stimulus current, which is difficult to accurately calculate; however, using an approximation method, and ignoring the spatial difference on the membrane, it can be estimated as

(26) $${ı}_{\text{s}}=\frac{V_{\text{s}}-V_{\text{m}}}{R_{s}\cdot S},$$

where *R*_s_ is the resistance of the pipette filled with solution, *V*_s_ is the electrode voltage, and *S* is the superficial area of the cell. If the location of the patch clamp is taken into account, the point on the membrane close to the hole pierced by the patch clamp is much more stimulated. With Equation 6, the ionic flux of stimulation can be estimated.

## 3. Ion Channel Model

Using the analysis above, one can see that the membrane potential is actually a measured value of the membrane ion density. Therefore, voltage-sensitive ion channels are essentially sensitive to the ionic density on the membrane surface. To maintain the ion concentration, ion channels are selective and sensitive to ions. Especially considering the evolutionary history of ion channels, we speculate that ion channels have a general functional mechanism . Thus, it is necessary to establish a general ion channel model to describe the passive transport of ions across cell membranes. Here we set three *basic assumptions* based on the discussion above:

**Assumption 1**. *The primary role of ion channels is to maintain specific intracellular ionic concentration*.

**Assumption 2**. *Ion channels can only detect ions on the inner surface of the membrane and the ions flowing through it*.

**Assumption 3**. *Ion channels can filter ions, but the flow of ions is passive*.

The transmembrane passive ionic flux is related to the ionic density difference and the selective permeability of ion channels. In a membrane, considering channels of the type *h* as a whole (called channel *h*), the ionic flux depends on its selective permeability and the ionic density difference,

(27) $$-\zeta^{\prime }=\rho^{\prime }\circ Dc,$$

where **ρ** = (ρ_*h, i*_), ρ_*h, i*_ is the selective permeability of channel *h* for ion *i*, **ζ** = (ζ_*h, i*_), ζ_*h, i*_ is the influx of ion *i* through channel *h* and

(28) $$D=T_{\text{in}}-T_{\text{ex}}.$$

Thus, the transmembrane ionic influx is

(29) $$\text{u}=\sum \zeta +{\text{J}}_{\text{s}}$$

if ion pump is absent, where **ȷ**_s_ is the stimulation ion influx. As generally understood, a channel can be abstracted as a combination of filter and gate. The filter that selects the ions is either conductive or inactivated, and the gate is either closed or open. Therefore, it can be designed that

(30) ρ=f∘g∘ϱ,

where ***f*** = (*f*_*h, i*_), *f*_*h, i*_ describes the filter of channel *h* to ion *i*, *g*_*h*_ describes the gate of channel *h*, and **ϱ** = (ϱ_*h, i*_), ϱ_*h, i*_ is the standard selective permeability of channel *h* to ion *i*.

### 3.1. Sensor

Generally, the intracellular ion concentration is stable. It is assumed (Assumption 2) that the ion channel can only detect the concentration of ions on the inner surface of the membrane. It is reasonable to assume that each type of ion channel has a specific internal ion density at which it is most comfortable. Assumptions 1 and 2 require that the ion channels are sensitive to the specific ions. For each channel *h*, the internal ionic density detected, (named *detected ionic density* in this paper written as σ_*h*_), depends on the internal ionic density and the sensitivity *s*_*h, i*_ to ion *i*. Assuming that the dependency is linear, then

(31) $$\sigma =T_{\text{in}}c\cdot s^{\prime },$$

and mark $\bar{\text{σ}}$ for optimum, named *optimum density*. In the same way, with the sensitivity **s**, channel *h* detects the ionic flux by

(32) $$\psi_{h}=\zeta_{h}\cdot s_{h},$$

named *detected ionic flux* in the same manner. We also know that the sensitivity **s** does not have to be proportional to the permeability **ϱ**. To be specific, channel *h* may be sensitive to ion *i* but allows ions *j*(≠*i*) to pass through. To facilitate the modeling below, we have designed two virtual indicators to describe the tendency of detected ionic density to deviate from the optimal density for ion channels — ϑ to point out the difference between detected ionic density and optimal density, and ϕ to point out the influence of detected ionic flux on density. According to usual practice, ϑ points to 0 for the optimum, as $\sigma /\bar{\sigma }=1$; −π/2 and π/2 for the worst, as $\sigma /\bar{\sigma }=0,\infty$; ϕ points to 0 for the optimum, as ψ/σ = 0. To satisfy these conditions, it is natural to state that

(33) $$\vartheta =2\text{atan}\sigma ⊘\bar{\sigma }-\frac{\pi }{2},$$

and

(34) $$\phi =\tau \circ \psi ⊘\bar{\sigma },$$

where **τ** = (τ_*h*_), τ_*h*_ is the empirical time constant of channel *h*. The larger τ_*h*_ is, the more stringent the restriction of gate on ion flow is.

### 3.2. Gate

The gate of the ion channel is a switch that controls the passive transportation of the intracellular and extracellular ions. According to the basic assumptions, an ion channel whose primary role is to maintain the intracellular ionic concentration, can only detect ionic density on the inner surface of the membrane and the flow of ions through it. When the detected ionic density is optimal, the ion channel *h* needs to keep this state and the gate should be closed. When the detected ion density deviates from the optimal density, $\sigma_{h}\neq {\bar{\sigma }}_{h}\Rightarrow \vartheta_{h}>0$, the ion channel needs to transfer ions. Given that this transportation is passive, the result may be better or worse. Thus, the opening and closing of the gate should be controlled by two parts,

(35) g=v∘w.

One is to explore and the status is measured by *v*_*h*_∈[0, 1]. As assumed before, *v*_*h*_ → 0 for ϑ_*h*_ = 0, *v*_*h*_ → 1 for $\vartheta_{h}=\pm \frac{\pi }{2}$. Then we can obtain geometrically that

(36) $$\frac{\text{d}}{\text{d}t}v=\alpha \circ (\bar{v}-v),$$

where the limiting value

(37) $$\bar{v}=1-\text{cos}\cdot^{\;\eta \;}\vartheta ,$$

**α** = (α_*h*_), α_*h*_ describes the agility of exploring of channel *h*; and **η** = (η_*h*_), η_*h*_∈(0, ∞) is the tolerance of channel *h* to ionic density deviation, describes the degree of which the channel allows the ion density to deviate from the optimal value. The value of η_*h*_ is empirical and uniformly set as 1 in this paper for each *h*. The other is to judge and is measured by *w*_*h*_∈[0, 1]. While *v*_*h*_>0:

- if σ_*h*_ approximates to the optimum, as *ϕ*_*h*_/ϑ_*h*_ < 0, the judgement is to keep the gate open;
- if σ_*h*_ deviates from the optimum, as *ϕ*_*h*_/ϑ_*h*_>0, the judgement is to close the gate;
- otherwise, as *ϕ*_*h*_/ϑ_*h*_ = 0, for random is the vibration of ions, *w*_*h*_ → 1/2 from the viewpoint of probability.

The execution of judgment still needs a process, and the bigger *v*_*h*_ is, the more urgent the process is. Thus, we state that

(38) $$\frac{d}{\text{d}t}w=\beta \circ (\bar{w}-w)\cdot (v+\delta ),$$

of which the objective is

(39) $$\bar{w}=\frac{1}{\pi }\text{acot}\phi ⊘\vartheta$$

to satisfy the above conditions about **w**, where **β** = (β_*h*_), β_*h*_ describes the agility of judgment of channel *h*, **δ** = (δ_*h*_), δ_*h*_ is positive, empirical, and constant on *h*, then $\beta_{h}\delta_{h}\left({\bar{w}}_{h}-w_{h}\right)$ is the rate of change of channel *h* while *v*_*h*_ = 0. If the detected ionic densities of all the channels on a membrane are optimal or very close to the optimum, then the membrane is in what is called a *resting state*.

### 3.3. Filter

It is generally acknowledged that ion channels filter the ions, like having a *filter*. When an ion channel is continuously stimulated, the filter changes the permeability of the ions. For example, application of a specific stimulus opens the intracellular gate of a potassium channel (activation), yielding a transient period of ion passive conduction until the selectivity filter spontaneously undergoes a conformational change toward a non-conductive state (inactivation). Removal of the stimulus closes the gate and allows the filter to interconvert back to its conductive conformation (recovery) (Ostmeyer et al., 2013). In addition, there is a remarkable correlation between the degree of gate opening and the conformation and ion occupancy of the selectivity filter (Cuello et al., 2010). For instance, the hERG channel opens when it is stimulated specifically and then gradually inactivates, but still allows the sodium to pass through in its specific inactive state (Gang and Zhang, 2006). In conclusion, we may consider that the filter occupied by the ion flow may gradually inactivate under continuous stimulation. It is inferred that ${\bar{f}}_{h,i}$ decreases with the stimulus intensity (measured by $\vartheta_{h}^{2}$) and detected ion flux (measured by $\phi_{h}^{2}$). Following the description of the filter state above, set ${\bar{f}}_{h,i}=1$ at *ϕ*_*h*_ = 0 and ϑ_*h*_ = 0. The model of filter can then be designed as

(40) $$\frac{d}{\text{d}t}f=\gamma \circ \left(\bar{f}-f\right),$$

(41) $$\bar{f}=\text{exp}\left(-\kappa \circ \phi^{\;\circ \;2m}\circ \vartheta^{\;\circ 2n}\right),$$

**γ** = (γ_*h*_), **κ** = (κ_*h, i*_), where γ_*h*_ and $\kappa_{h,i}\in {\mathbb{R}}^{+}$, *m*_*h, i*_ and *n*_*h, i*_∈ℕ are experiential, γ_*h*_ describes the agility of the filter, and κ_*h, i*_ is the inactivation coefficient of channel *h* occupied by ion *i*.

## 4. Results

In *Foundation*, we have introduced how ionic flux affects ion concentration, and how to calculate membrane potential from ionic concentration and membrane current from passive transmembrane ionic flux. We then constructed a *ion channel model*, which can be used to simulate how the ion distribution affects the passive transmembrane ion flux. Thus, we may compare the numerical simulation results with the measured experimental data to validate the model and discuss its nature.

Although cells are different in shape and size, given our focus on the properties of membranes and channels, this does not affect the analysis of the model to unify the cells into a sphere with radius *r* = 10 μm, especially for ion channels.

### 4.1. Estimation of Capacitance

**Figure 1:**
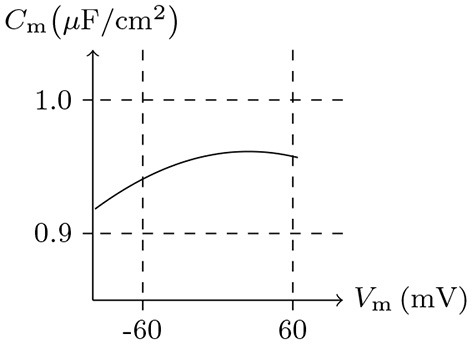
Numerical simulation of membrane capacitance. Relative permittivity (as default in this paper): ε_r_ = 4. Extracellular solution: (i). Intracellular solution: (ii). It shows that the membrane capacitance is not symmetrized to *V*_m_ = 0 mV — a phenomenon is common in all kinds of media (Hamelin et al., 1983).

Here we use the method outlined in *Foundation* to calculate the membrane capacitance and compare it with the actual measurement. The detailed method is to calculate the membrane potential and internal charge first and then to estimate the membrane capacitance by *C*_m_ = d*Q*/d*V*_m_. To simulate a real environment and close all the channels, given common $\left(\begin{array}{c} A_{\text{ex}}\\ A_{\text{in}} \end{array}\right)$***c*** and *ı*_s_, the relationship between intracellular electric charge *Q* and membrane potential *V*_m_ will be obtained, with which the membrane capacitance *C*_m_ with the potential can then be calculated. In reality, the relative permittivity of cytomembrane ε_r_ is generally between 1.4 and 20 (Zhang et al., 2014). Let ε_r_ = 4, for *V*_m_/mV∈(−100, 100), the estimated membrane capacitance $C_{\text{m}}\approx 1\text{ μF}/\text{c}{\text{m}}^{2}$, is consistent with the credible measured values of giant axons (Hodgkin et al., 1952); see Figure 1.

### 4.2. Gate Properties

This subsection mainly analyses the nature of the inner gate in the model, which can be verified by the comparison of digital simulations and biological experiments.

#### 4.2.1. Potassium Channel From Plant Root Cell

**Figure 2:**
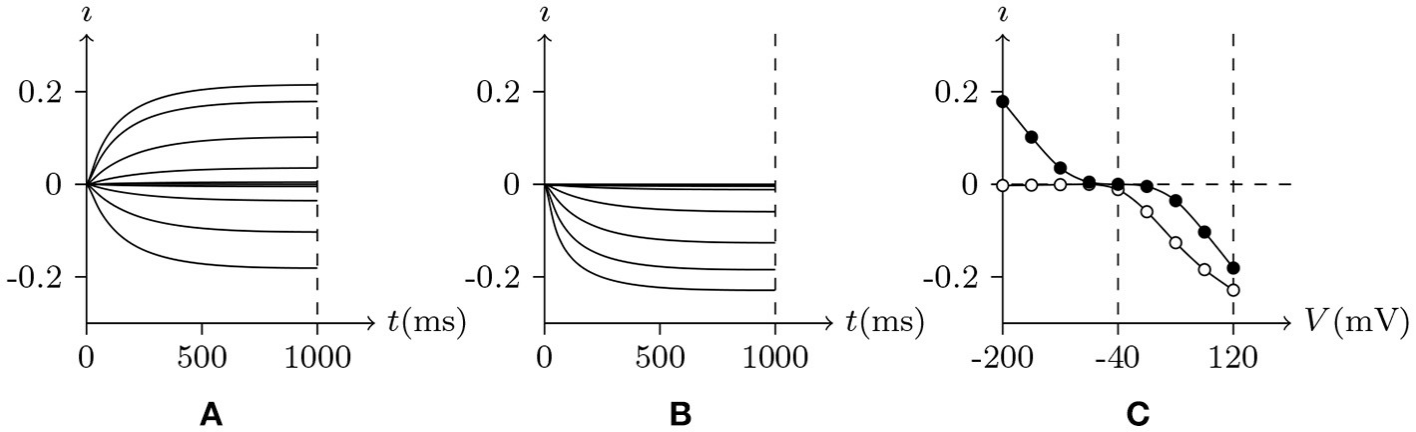
Numerical simulation current I(A/m^2^) of potassium channels in wild-type root cells. Channel list: K_v_♣. Patch clamp: *R*_s_ = 2.4 MΩ; *V*_s_ = −40 mV for *t*/ms < 0, *V*_s_ = −200 to 120 mV for *t*/ms>0. Intracellular solution: (iv). Extracellular solution: (v) for **(A)**, (vi) for **(B)**. The points in **(C)** are the values of current recorded at *t* = 1s, black for **(A)**, white for **(B)**.

First simulate a plant root cell potassium channel by our model, of which the phenomenon is seen in plant root potassium channel electrophysiological experiments: if the external solution is rich in potassium ions, the hyperpolarization of wild-type root cell produces an inward current, which disappears when extracellular K^+^ is replaced by Cs^+^ (Hirsch et al., 1998). The numerical simulation is shown in Figure 2. Based on the properties of the model, in resting state:

- Holding *V*_s_ > *V*_∞_, the intracellular cations move to the inner surface of the membrane and the extracellular cations leave the outer surface. Then the inner surface potassium density is higher than the density in resting state, $\sigma >\bar{\sigma }\Rightarrow \bar{v}>0$, and the potassium channel will be open. As an experimental environment in general, the inner surface potassium density is greater than the outer surface, ζ < 0, so $\bar{w}>1/2$, and the gate will stay open.
- Holding *V*_s_<*V*_∞_, the intracellular cations leave the inner surface of membrane and the extracellular cations move to the outer surface. Then the inner surface potassium density is lower than the density in resting state, $\sigma <\bar{\sigma }\Rightarrow \bar{v}>0$, and the potassium channel will also be open.
  - If the extracellular potassium ion is scarce, $\frac{{\left[{\text{K}}^{+}\right]}_{\text{ex}}}{{\left[{\text{K}}^{+}\right]}_{\text{in}}}\approx 0$, the intracellular potassium will be transported passively to the outside of the cell, then $\bar{w}\approx 0$, and the gate of the potassium channel will be closed to stop the potassium outflow.
  - Otherwise, if the external potassium concentration is high enough, ϑ < 0 and *ϕ*>0 ⇒ $\bar{w}>1/2$, and the potassium channel will stay open.

#### 4.2.2. Sodium Channel and Refractory Period

**Figure 3:**
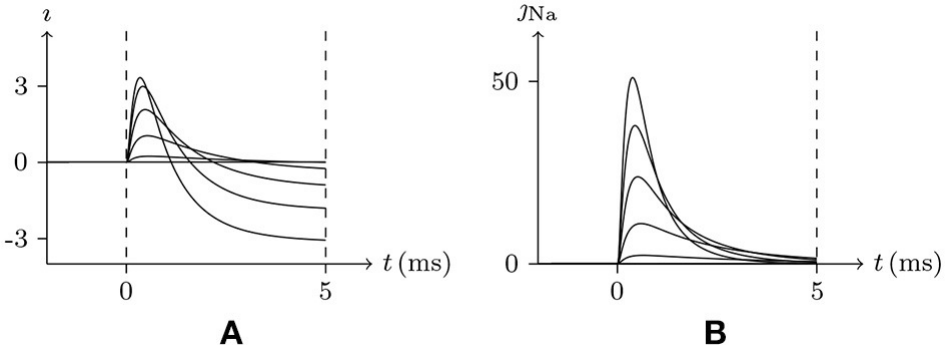
Numerical simulation from activation to inactivation of sodium channel. **(A)** shows the membrane current, of which the unit is A/m^2^, generated by sodium, potassium, and chloride ion flux; **(B)** the sodium influx, the unit is μmol/s/m^2^. Channel list: Na_v_♠, K_v_♠, Cl_v_♠. Patch clamp: *R*_s_ = 2.4 MΩ; *V*_s_ = −70 mV for *t*/ms ≤ 0, *V*_s_ = −70 mV to 30 mV for *t*/ms>0. Extracellular solution: (i). Intracellular solution: (ii).

**Figure 4:**
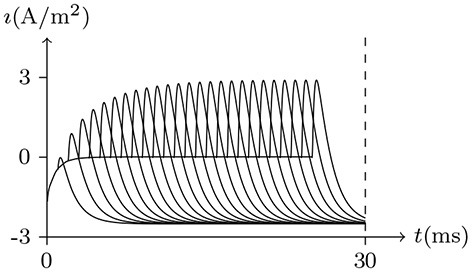
Numerical simulation of recovery from refractory period. Patch clamp: *V*_s_ = −70 mV for *t*/ms∈[0, Δ*t*], *V*_s_ = 30 mV for *t*/ms∉[0, Δ*t*], Δ*t* = 0–25. The other settings are the same as in Figure 3. With the Δ*t* increasing, the sodium channel gradually revives to the resting state as the end of the refractory period.

In common patch clamp experiments, the intracellular solution is rich in potassium ions, and the sodium ion concentration is extremely low. Therefore, the sodium channel is a good contrast with the potassium channel for discussing the properties of the gate. In sodium channel experiments, the external solution is generally rich in sodium ions, ${\left[\text{N}{\text{a}}^{+}\right]}_{\text{in}}\ll {\left[\text{N}{\text{a}}^{+}\right]}_{\text{ex}}$. In such an environment, when stimulating the membrane, the sodium channel opens briefly and then enters the refractory period. This phenomenon can be seen in the early experiment on the giant axon of the Loligo (Hodgkin and Huxley, 1952), of which the numerical simulation result is shown in Figure 3. After the stimulus is removed, the sodium channel will gradually recover. This phenomenon has been well documented in the study of excitable membranes (Hille, 2001), of which the numerical simulation result by our model is shown in Figure 4. By the model's calculation, in resting state:

1. Given *V*_s_ > *V*_∞_, the sodium ion density on the inner surface is higher than the resting state, $\sigma >\bar{\sigma }\Rightarrow v\to \bar{v}>0$, and the sodium gate should be open as the channel is activated. However, when the external sodium ion concentration is much greater than the internal, as the gate is opening, the sodium ions will flow backward, $w\to \bar{w}\approx 0$, and the gate will close again. In the case that α is large enough, such as α/β>0.1, the gate opens extremely fast. In the time between the opening and closing of the gate, there is a short but significant sodium ion influx. As $w\to \bar{w}\approx 0$, the sodium gate tends to be closed, the channel inactivates, and the refractory period commences.
2. Once the stimulus is eliminated, $\bar{w}\approx 1$, the channel will gradually recover. Subsequent *w*≈1 means the end of refractory period.

### 4.3. Filter Properties

It was easy to infer from the concentration of the external solution that the outward current initially found on nerve cells was generated by the potassium ion flow. Based on the axon experiments, it was believed that the potassium channels would not be closed before the depolarization stimulus was removed. However, in the subsequent experiments, transient outward potassium current was observed.

#### 4.3.1. Transient Outward Potassium Channel

**Figure 5:**
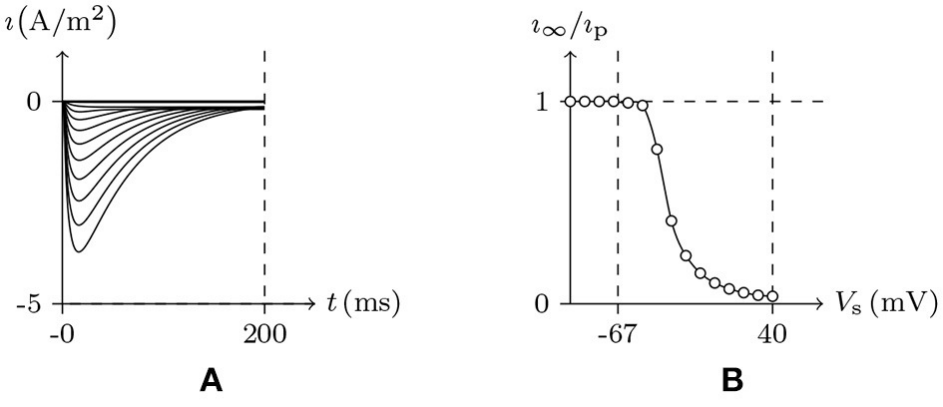
Numerical simulation of transient outward potassium current. **(A)** Activation and inactivation curves. **(B)** Steady-state inactivation curves. Channel list: K_v_4.3. Patch clamp: *R*_s_ = 2.4 MΩ; *V*_s_ = −70 mV for *t*/ms ≤ 0, *V*_s_ = −100–40 mV for *t*/ms>0. Extracellular solution: (i). Intracellular solution: (ii).

**Figure 6:**
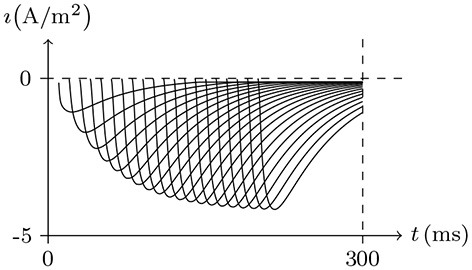
Recovery from inactivation of transient outward current by numerical simulation. *V*_s_ = 50mV for *t*/ms∈(−∞, 0)∪(Δ*t*, ∞), *V*_s_ = −70mV for *t*/ms∈[0, Δ*t*], Δ*t* = 0 to 200. The other settings are the same as in Figure 5.

The typical phenomena of transient outward current can be seen in experiments conducted on neural somata (Connor and Stevens, 1971) and cardiomyocytes (Akar et al., 2004), of which the numerical simulation results by our model are shown in Figures 5, 6.

As we have mentioned above, the conformation of the selectivity filter is correlated to the degree of gate opening, and more specifically, depends on the detected stimulation intensity φ and the occupation *ϕ* by the ions. κ determines the type of ion channel inactivation. Based on the model calculation, in the environment of ${\left[K^{+}\right]}_{ex}/{\left[K^{+}\right]}_{in}\approx 0$, given β≫α≫κ:

- In the case of *V*_s_ < *V*_ ∞ _, $\sigma <\bar{\sigma }$. For the reason that S_in_*c*_*K*_>S_ex_*c*_*K*_ $\left(⇐{\left[K^{+}\right]}_{ex}/{\left[K^{+}\right]}_{in}\approx 0\right)$, as the gate is opening, the potassium ion outflow leads to $w\to \bar{w}\approx 0$, and the gate will be closed. For β ≫ α ≫ κ, the gate can be considered to always be closed so that the filter state remains constant.
- In the case of *V*_s_>*V*_∞_, the gate will be open and will expand for the duration of the stimulation. For α ≫ κ, the change of *f* lags behind *g*. The potassium current will first increase significantly and then decrease. The peak increases with the stimulation. See Figure 5. After the stimulus is removed, the gate will be closed again and the inactive filter will gradually recover to the resting state. The longer the interval between two stimuli is, the closer the second peak is to the first; see Figure 6.

#### 4.3.2. Delayed Rectifier Potassium Channel

**Figure 7:**
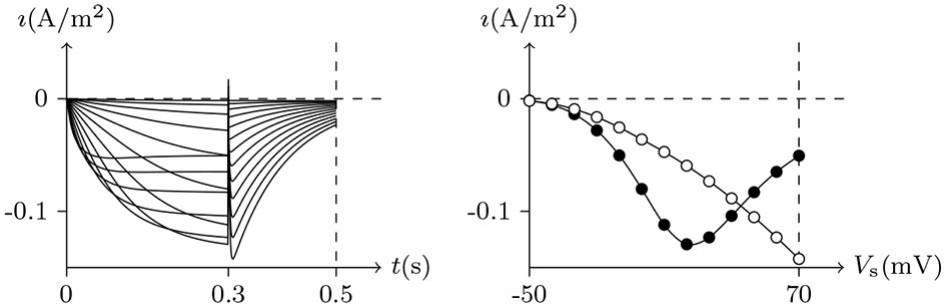
Numerical simulation current of hERG. Channel list: hERG. Patch clamp: *R*_s_ = 2.4MΩ, *V*_s_ = −70mV for *t*/ms ≤ 0, *V*_s_ = −50mV to 70mV for *t*/ms∈(0, 300], *V*_s_ = −50mV for *t*/ms>300. Extracellular solution: (i). Intracellular solution: (ii). The black points are recorded at *t* = 0.3s, the white points are the peaks of the tail currents in (0.3, 0.5) of *t*/s.

**Figure 8:**
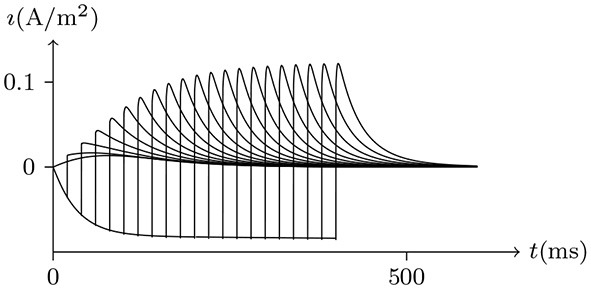
Activation current of hERG by numerical simulation. *V*_s_ = −70mV for *t*/ms ≤ 0, *V*_s_ = 50mV for *t*/ms∈(0, Δ*t*), *V*_s_ = −150mV for *t*/ms≥Δ*t*, Δ*t* = 0 to 400. The other settings are the same as in Figure 7.

As we have said earlier, κ determines the type of ion channel inactivation. In contrast to the transient outward potassium channel, is the rectifier potassium channel. The typical phenomena can be observed in an experiment conducted on hERG (Ficker et al., 1998), of which the numerical simulation by our model are shown in Figures 5 , 6. By the model calculation, given κ ≫ α:

1. Holding *V*_s_>*V*_∞_, the channel activates slowly on moderate membrane depolarization and exhibits limited outward current at a more positive membrane depolarization. After the membrane current is relatively stable, on return to negative membrane potentials, removal of C-type inactivation results in an initial strengthening in tail currents with slow deactivation kinetics, for $\frac{\text{d}f}{\text{d}t}\gg -\frac{\text{d}g}{\text{d}t}$. The stronger the previous depolarization, the stronger the tail current. See Figure 7.
2. At a certain point in time after the outward current is relatively stable, the switch of *V*_s_ from depolarization to hyperpolarization of *V*_s_<*V*_∞_ will generate an instant inward current. The longer the depolarization lasts, the stronger the instant inward current is. See Figure 8.

Transient outward and delayed rectifier potassium channels are common in cardiomyocytes, of which the numerical simulation shows a general applicability to our model. We have analyzed the relationship between the filter properties and the types of ion channel inactivation within some typical cases.

### 4.4. Special Sensitivity

There are some cases we have given above in which sensitivity and permeability correspond incompletely ($\frac{{\text{ϱ}}_{h}}{\left||{\text{ϱ}}_{h}|\right|}\neq \frac{{\text{s}}_{h}}{\left||{\text{s}}_{h}|\right|}$). To be more extreme, it is an absolute necessity to consider whether there are some channels particularly sensitive to certain ions but very permeable to others. An affirmative answer to this question may indicate a dual dependence on specific ion concentration and voltage of these channels. In reality, calcium-activated potassium channels are common and sensitive to calcium ions but permeable to potassium. Since all the channels mentioned above are sensitive and transparent to cations, we are naturally led to ask whether there are some ion channels sensitive to cations but permeable to anions. The calcium-activated chloride channel (CACC) is a good example.

#### 4.4.1. Calcium-Activated Chloride Channel

Let us assume that a certain type of channel can be very sensitive to calcium ions but permeable to chlorine, and let $\frac{{\left[\text{C}{\text{a}}^{2+}\right]}_{\text{ex}}}{{\left[\text{C}{\text{a}}^{2+}\right]}_{\text{in}}}\approx 0$.

- If ${\left[\text{C}{\text{a}}^{2+}\right]}_{\text{in}}$ is low enough to satisfy $\sigma \ll \bar{\sigma }$ in the initial state, then $w=\bar{w}\approx 0\Rightarrow g=\bar{g}\approx 0$, and the gate will remain almost closed under any stimulation.
- If ${\left[\text{C}{\text{a}}^{2+}\right]}_{\text{in}}$ is moderate, $\sigma \approx \bar{\sigma }\Rightarrow \bar{g}\approx 0$ for *V*_s_ = *V*_∞_, the channel is almost closed for *V*_s_ = *V*_∞_, but open for *V*_s_≠*V*_∞_ because of $\sigma \neq \bar{\sigma }$ under this circumstance.
- If ${\left[\text{C}{\text{a}}^{2+}\right]}_{\text{in}}$ is high enough to satisfy $\sigma \gg \bar{\sigma }$ for *V*_s_ = *V*_∞_, easily derived $\bar{g}>0$ in this circumstance, the channel is already open before the stimulus.

**Figure 9:**
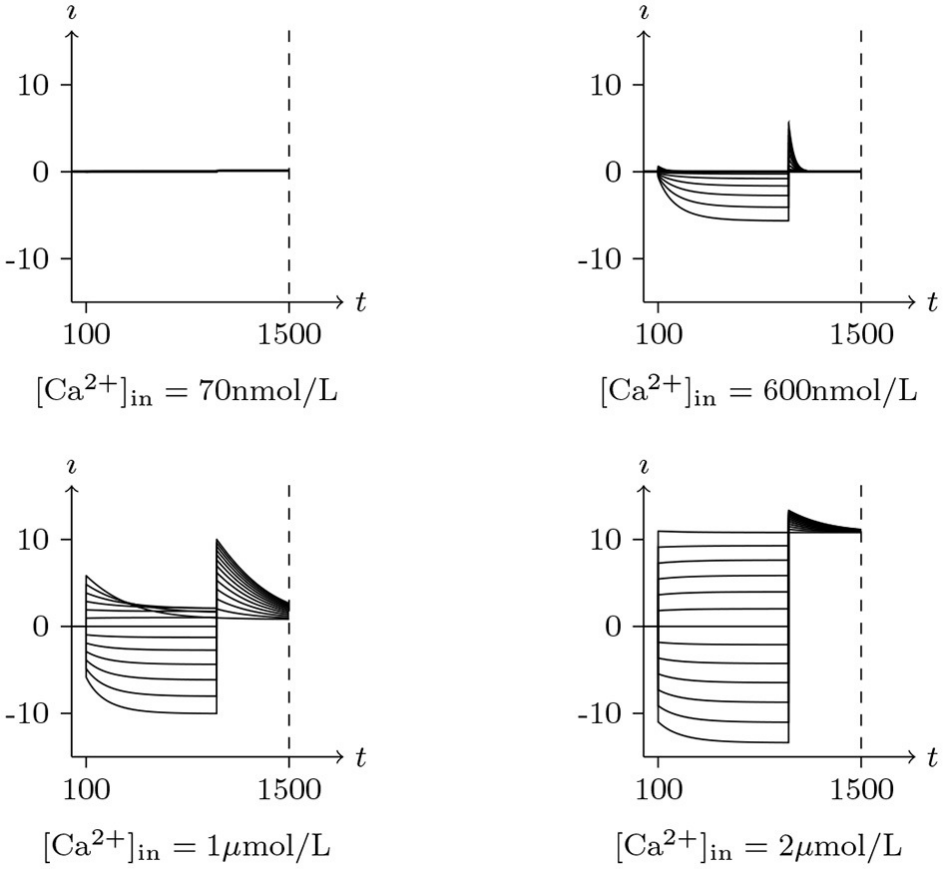
Numerical simulation activation of CACC currents Channel list: CACC. Patch clamp: *R*_s_ = 4.0MΩ, *V*_s_ = 0mV for *t*/s < 0.1, −120mV to 120mV for *t*/s∈(0.1, 1], −120mV for *t*/s>1. Intracellular and Extracellular solution: (vii), using Ca-EGTA to control ${\left[\text{C}{\text{a}}^{2+}\right]}_{\text{in}}$. The unit of I is A/m^2^, and the unit of *t* is ms.

**Figure 10:**
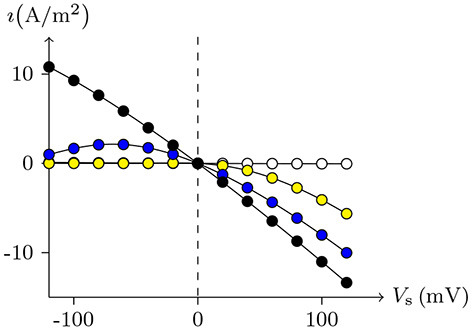
Numerical simulation steady state current-voltage relationships of CACC. The values of electric current density recorded at *t* = 1s in Figure 9, *V*_s_ from −120mV to 120mV, white for 70 nmol/L, yellow for 600 nmol/L, blue for 1μmol/L, black for 2μmol/L.

Therefore, this type of chloride channel has a dual dependence on membrane potential and intracellular calcium concentration, of which the peculiarities, shown in Figures 9,10, are very consistent with the CACC (the samples were taken from xenopus oocytes) that play fundamental roles in physiological processes in many tissues (Kuruma and Hartzell, 2000).

### 4.5. Combined Effect of Channels

It is well-documented that there is more than one type of ion channel in a cell membrane. In our model, the ion channels are responsible only for the density of specific ions on the inner surface of the membrane. The state change of the ion channel depends on the ionic density on the inner surface of the membrane, such that there is no direct correlation among the ion channels. However, because the opening of one ion channel affects the distribution of ion concentrations in the membrane and thus affects the states of other ion channels, the ion channels on the same membrane interact with each other via ions, and produce a variety of complex electrophysiological phenomena, such as the nerve impulse, which is a result of the combined effect of the ion channels. In general, in experimental environments, extracellular fluid is rich in sodium, ${\left[\text{N}{\text{a}}^{+}\right]}_{\text{in}}<{\left[\text{N}{\text{a}}^{+}\right]}_{\text{ex}}$, intracellular fluid is rich in potassium, ${\left[{\text{K}}^{+}\right]}_{\text{in}}>{\left[{\text{K}}^{+}\right]}_{\text{ex}}$, and both are rich in chlorine, which is also the main electrode reactant. Thus, from the numerical model standpoint, the combination of sodium, potassium, and chlorine channels should be relatively sufficient; this is exactly the general structure observed in neurons.

#### 4.5.1. Nerve Impulse

There exist several simple methods to observe changes in action potentials. From the perspective of our model, let us analyse the process of neural excitation.

**Figure 11:**
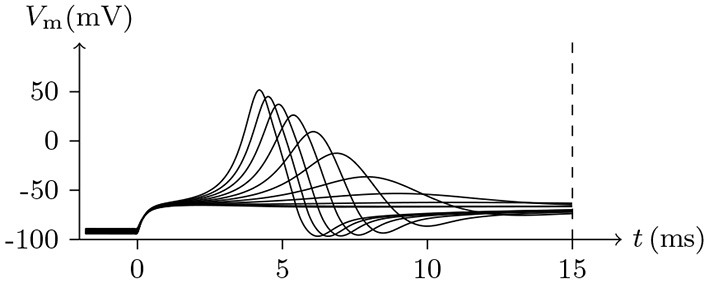
Numerical simulation nerve action potential of anode break excitation following sudden cessation of external current. Channel list: Na_v_♠, K_v_♠, Cl_v_♠. Stimulus current: $I_{\text{s}}/\text{A}/{\text{m}}^{2}=-0.6$ to −0.9 for *t*/ms∈[−50, 0], 0 for *t*/ms>0. The other settings were the same as in Figure 3.

**Figure 12:**
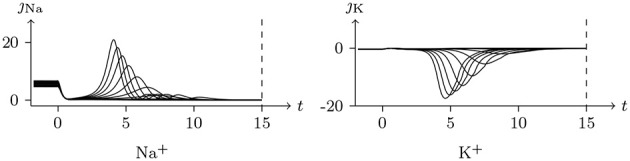
Numerical simulation transmembrane ionic flux of anode break excitation following sudden cessation of external current in the nerve. The transmembrane ionic fluxes (μmol/s/m^2^) of sodium and potassium with time (ms) are shown. The settings were the same as in Figure 11.

Holding *V*_s_ < *V*_∞_, based on the analysis above, it can be concluded that the sodium and chlorine channels are open, whereas the potassium channel is almost closed. As the voltage clamp is released, the closure of the sodium and chlorine channels will take a short time. It takes a transient stimulus to produce a potential spike (see Figure 11), which is generated conjointly by three different channels (see Figure 12). The corresponding classic experimental phenomena were recorded in an earlier paper (Hodgkin and Huxley, 1952).

Given a depolarized stable $I_{\text{s}}$, as mentioned, the sodium ion channel will be open briefly. In this process, the membrane potential will increase sharply and the potassium and chloride ion channels will also be opened to make the membrane potential fall back down again.

**Figure 13:**
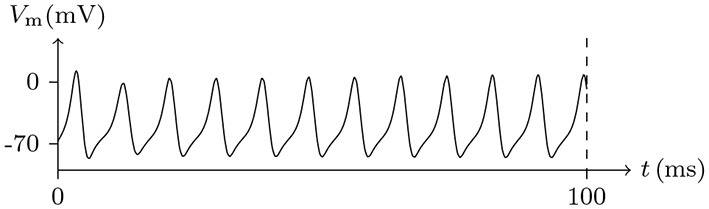
Numerical nerve action potential of periodic spiking. The constant direct current stimulation: $I_{\text{s}}=0.1\text{A}/{\text{m}}^{2}$. The other settings were the same as in Figure 3.

**Figure 14:**
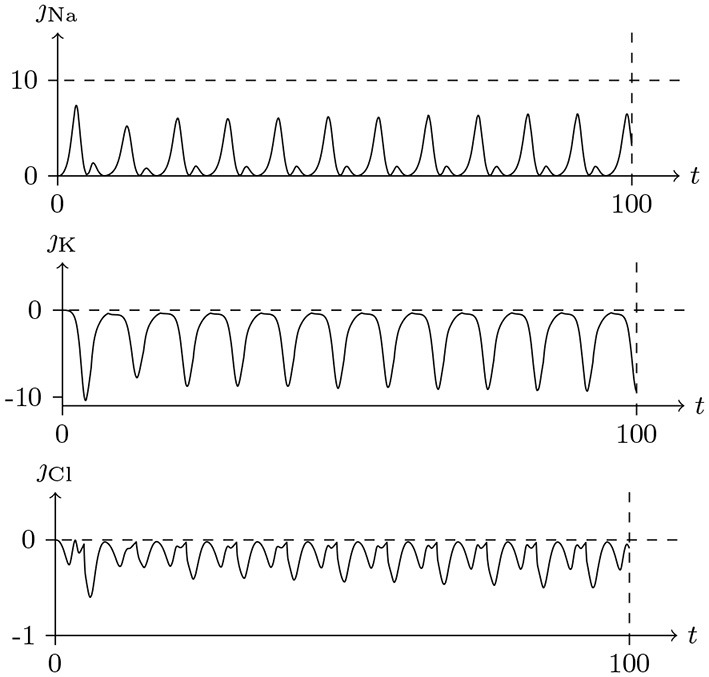
Numerical simulation transmembrane ionic flux density of periodic spiking. The numerical ion fluxes (μmol/s/m^2^) of sodium, potassium and chloride with time (ms) are shown in order. The settings were the same as in Figure 13.

**Figure 15:**
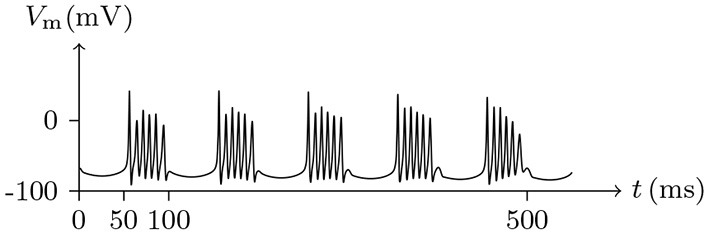
Numerical simulation nerve action potential of bursting. The alternating current stimulation: $I_{\text{s}}=0.2\text{A}/{\text{m}}^{2}\cdot \sin20\pi t/\text{s}$. The other settings were the same as in Figure 13.

- If the depolarizing direct current stimulus remains at an appropriate intensity, the channels may be excited repeatedly, forming periodic spiking (Ram-rez Piscina and Sancho, 2018), of which the numerical stimulation is shown in Figures 13, 14.
- If the alternating current stimulation is given with appropriate frequency and intensity, then bursting is generated (Figure 15).

The parameters of the sodium, potassium, and chloride ion channels in the model determine the frequency of periodic spiking. By comparing Figure 13 with 14, it is clear that the oscillations of ionic fluxes are much more complex than the membrane potential.

## 5. Discussion

In this paper we attempt to establish a general model of ion passive transmembrane transport based on ion concentration that conforms to the general understanding of ion channel today. The model is simple but capable of explaining and simulating the complex phenomena of patch clamp experiments, and is universally applicable to different ion channels in the different cells of different species.

In experiments, it has been found that many kinds of stimulation can produce electrophysiological phenomena, and the action of ion channels also seems to depend on a variety of external factors. These phenomena make the properties of ion channels so difficult to understand that some people believe that different models must be built to explain the different phenomena.

For example, a model based on the cooperative activation of sodium channels that reproduces the observed dynamics of action potential initiation to simulate the features of the initiation dynamics of cortical neuron action potentials has been proposed (Bjorn et al., 2006). However, it was shown that these features are to be expected from the model of Hodgkin and Huxley, if the spatial geometry and spike initiation properties of cortical neurons are taken into account. It is therefore unnecessary to invoke exotic channel-gating properties as an explanation (McCormick et al., 2007).

We believe that building different models for different phenomena, without explaining the commonality by a general model, will not help us further our understanding. It has been demonstrated countless times that the closer a model gets to the truth, the simpler it becomes. When we combine the factors which affect the ion channels, it is natural to establish a model based on membrane surface ion density. In this way, all the ion channels in this paper have been described by this general model, of which the numerical simulation results are consistent with the electrophysiological phenomena, universal to plant cells, oocytes, myocytes, cardiomyocytes, and neurocytes. We chose these experiments not only based on cell types, but also because their phenomena are representative.

These results suggest that when ion channels are responsible for intracellular ionic concentration, they are sufficient to produce a variety of electrophysiological phenomena.

## 6. Vista

### 6.1. Pump

Without the action of the pump, the intracellular ion concentration that deviated from the resting state due to passive transmembrane transport would not completely return to initial levels. A common model for the pumps has been built in our laboratory, though not yet validated by precise experimental data. The combination of ion channels and pumps constitute the complete transmembrane ion transport, which we will implement in the future. Once this is completed, our model will explain the process of intracellular ion concentration formation and maintenance.

### 6.2. Conduction

In this paper, the ionic flux is perpendicular to the membrane surface. To add the ionic flux parallel to the membrane surface, the complete picture of ionic flux will be calculated, especially for dendrites and axons. The early models only focus on the internal and transmembrane current or ionic flux of cells, generally. However, it is not difficult to deduced that an external ionic flux also exists. Further, in dendrites and axons, the direction of ion flow can be divided into transmembrane and axial and the axial ion flow is sensitive to the axial electric field. So the effect of the outer electromagnetic field is a very important subject, which needs to be further studied. Also, our model may extend to the connection between cells; for example, connections between nerve and nerve (synapse) and nerve and muscle (neuromuscular junction).

### 6.3. Information

Ion channels are sensitive to specific ions and control the passive transmembrane transport of ions. Therefore, the ionic flux and concentration can be regarded as the signals of cell state. From ionic density to voltage and ionic flux to electric current, there is a loss of information, which is hindering the exploration of the human brain. The artificial neural network theory based on the classical model is also inherently flawed. Our future research will try to distinguish and understand the ion signals of cells. On that basis, the establishment of a new artificial neural network model may contribute to the development of artificial intelligence.

## Author Contributions

VW: Conceptualization, software and writing; SL: Supervision.

### Conflict of Interest Statement

The authors declare that the research was conducted in the absence of any commercial or financial relationships that could be construed as a potential conflict of interest.

## Appendix

### A.1. Solution of Experiments

The solution of numerical experiments in this paper (in mmol/L): (i) NaCl 139, NaOH 1, KCl 5, CsCl 1, Ca-EGTA 1, MgCl_2_ 1; (ii) NaCl 9, NaOH 1, KCl 120, CsCl 1, Ca-EGTA 1, MgCl_2_ 2; (iii) NaCl 9, NaOH 1, KCl 120, CsCl 1, Mg-EGTA 1, MgCl_2_ 2; (iv) NaOH 1, KCl 40, CsCl 1, Ca-EGTA 1, MgCl_2_ 1, Sorbitol 90; (v) NaOH 1, KCl 1, CsCl 30, Ca-EGTA 1, MgCl_2_ 1, Sorbitol 120; (vi) NaOH 1, KCl 30, CsCl 1, Ca-EGTA 1, MgCl_2_ 1, Sorbitol 120; (vii) NMDG-Cl 139, NMDG-OH 1, KCl 5, CsCl 1, Ca-EGTA 0.001, MgCl_2_ 1.

### A.2. Ionic Radius

The ionic radiuses in this paper (pm): Na^+^ 102, Mg^2+^ 72, Cl^−^ 181, K^+^ 138, Ca^2+^ 100, Cs^+^ 167, OH^−^ 137.

### A.3. Parameters of the Channel Model

All the parameters of the channel model in this paper are recorded in Tables A1–A4 in Appendix. The parameters unassigned in appendix, defaults to 0.

**Table 1:** Filter state parameters.

***h***	**$\gamma_{h}\left({\text{ms}}^{-1}\right)$**	κ_*****h**, **i*****_					***m*_*h*_**	***n*_*h*_**
		Na^+^	K^+^	Cs^+^	Ca^2+^	Cl^−^		

K_v_♣ - plant root cell	0.1	-	1.0 × 10^−5^	-	-	-	1	-
K_v_4.3 - human heart cell	0.02	-	1.0 × 10^−11^	-	-	-	4	2
hERG - human heart cell	0.5	0.75	1.0	-	-	-	1	4

**Table 2:** Properties of gate and sensor.

***h***	**$\alpha_{h}\left({\text{ms}}^{-1}\right)$**	**$\beta_{h}\left({\text{ms}}^{-1}\right)$**	**δ_*h*_**	**${\bar{\sigma }}_{h}\left(nmol/m^{2}\right)$**	**τ_*h*_(s)**
K_v_♣ - plant root cell	4.0 × 10^−3^	3.0	1.0 × 10^−2^	9.6	1.0
K_v_♠ - loligo axon	0.5	50	5.0 × 10^−3^	25.0	10.0
Na_v_♠ - loligo axon	5.0	50	5.0 × 10^−3^	1.7	10.0
Cl_v_♠ - loligo axon	2.0	50	5.0 × 10^−3^	69.0	10.0
K_v_4.3 - human heart cell	0.2	50	5.0 × 10^−3^	25.0	10.0
hERG - human heart cell	0.01	0.5	1.0 × 10^−3^	25.0	1.0 × 10^2^
CACC - xenopus oocyte	5.0 × 10^−3^	3.0 × 10^−3^	10.0	1.0 × 10^−4^	1.0 × 10^5^

*The sensitivity and permeability parameters of inductor are shown in Tables 3, 4*.

**Table 3:** The values of sensitivity parameter *s*_*h, i*_.

	**Na^+^**	**K^+^**	**Cs^+^**	**Ca^2+^**	**Cl^−^**
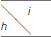
K_v_♣ - plant root cell	-	1	-	-	-
K_v_♠ - loligo axon	-	1	-	-	-
Na_v_♠ - loligo axon	1	-	-	-	-
Cl_v_♠ - loligo axon	-	-	-	-	1
K_v_4.3 - human heart cell	-	1	-	-	-
hERG - human heart cell	-	1	-	-	-
CACC - xenopus oocyte	-	-	-	1	-

**Table 4:** Standard selective permeability $\varrho_{h,i}\left({\text{ms}}^{-1}\right)$ of filter.

	**Na^+^**	**K^+^**	**Cs^+^**	**Ca^2+^**	**Cl^−^**
K_v_♣ - plant root cell	-	3.0	0.3	-	-
K_v_♠ - loligo axon	-	30.0	-	-	-
Na_v_♠ - loligo axon	1.5 × 10^2^	15	-	-	-
Cl_v_♠ - loligo axon	-	-	-	-	10.0
K_v_4.3 - human heart cell	-	50.0	-	-	-
hERG - human heart cell	2.4	4.0	-	-	-
CACC - xenopus oocyte	-	-	-	1.0 × 10^−2^	10.0
