# Corrigendum: A General Model of Ion Passive Transmembrane Transport Based on Ionic Concentration

**DOI:** 10.3389/fncom.2020.00048

**Published:** 2020-06-05

**Authors:** Vincent Qiqian Wang, Shenquan Liu

**Affiliations:** School of Mathematics, South China University of Technology, Guangzhou, China

**Keywords:** membrane, ion channel, gate, filter, ionic concentration and flux, electrophysiology, neurosciences

In the original article, we neglected to include the funder “the National Natural Science Foundation of China, 11572127 and 11872183” to “Shenquan Liu”. In addition, in the **Appendix**, subsection **A.1. Solution of Experiments**, we stated it was “MgCl_4_” when it should have been “MgCl_2._”

The authors apologize for this error and state that this does not change the scientific conclusions of the article in any way. The original article has been updated.

